# Evaluation of the Inactivation Efficacy of Four Disinfectants for Feline Parvovirus Derived from Giant Panda

**DOI:** 10.3390/microorganisms11071844

**Published:** 2023-07-20

**Authors:** Qianling Peng, Zhisong Yang, Lin Wu, Peilun Yu, Qiang Li, Jingchao Lan, Li Luo, Shan Zhao, Qigui Yan

**Affiliations:** 1 College of Veterinary Medicine, Sichuan Agricultural University, Chengdu 611130, China; 2 Institute of Giant Panda Science of Sichuan, Chengdu 610084, China; 3 Chengdu Research Base of Giant Panda Breeding, Chengdu 610081, China

**Keywords:** disinfectant, feline panleukopenia, FPV, giant panda, inactivation

## Abstract

Feline panleukopenia (FPL) is a highly contagious acute infectious disease caused by feline parvovirus (FPV). FPV has also been found in giant pandas with clinical signs of vomiting and mild diarrhea, posing a threat to this vulnerable species. Cleaning and disinfection may be one of the most efficacious ways to prevent FPV spread in the habitat of giant pandas. This study evaluated the inactivation effect of peracetic acid (PAA), povidone-iodine (PVP-I), glutaral and deciquam solution (JM) and Virkon S. The tissue culture infective dose (TCID_50_) assay indicated that the virus may be totally inactivated by JM, PAA and Virkon S. Meanwhile, the hemagglutination (HA) assay showed a high inactivation efficiency of PAA and Virkon S. The analysis of Western blot revealed that PAA, Virkon S and JM can inhibit the structural protein synthesis. Taken together, our findings demonstrated that PAA could rapidly and efficiently inactivate FPV, representing an efficacious disinfectant for FPV control.

## 1. Introduction

FPV (feline parvovirus), usually associated with FPL, is a member of the genus *Protoparvovirus* (order *Ortervirales*, family *Parvoviridae*, subfamily *Parvovirinae*) [1]. FPV has a wide host range and high virulence, and it has been reported to infect domestic cats (*Felis catus*), tiger (*Panthera tigris*) [2], mountain lions (*Puma concolor*) [3], linsang (*Prionodon linsang*) [4], monkey (*Primates*) [5], and even giant pandas (*Ailuropoda melanoleuca*). The giant panda, originating from China, is listed as ‘vulnerable’ by the International Union for the Conservation of Nature (IUCN 2021). Giant pandas face many threats, such as viral infection, environmental damage [6], parasitic infection [7,8] and toxic compounds [9]. There is evidence showing that an expanding habitat increases the possibility of cross-species transmission of viral pathogens, such as canine distemper virus (CDV) [10,11,12], canine coronavirus (CCV), canine parvovirus (CPV) [10,13], canine adenovirus (CAV) and H1N1 influenza A virus [14]. According to a report, an FPV isolate Giant panda/CD/2018 was obtained from a captive giant panda in China for the first time, which indicated a host jump from cats to giant pandas [15]. The clinical symptoms of giant pandas infected with FPV were vomiting and mild diarrhea [16]. Like CDV, CCV and CPV, FPV also threatens the health of giant pandas. Since there is no FPV vaccine specific for giant pandas, the prevention of FPV is important. Given the contact transmission and resistance to many disinfectants, preventing infection of FPV requires practicable disinfection.

FPV spreads by direct or indirect contact with the feces, urine and blood of infected animals. Thus, keeping the host animals’ living environment clean is of great importance to prevent them from being infected with FPV. However, without thorough disinfection, environmental contamination can remain infectious for many months [17]. FPV possesses resistance to many commonly used disinfectants, but it can be inactivated by products containing potassium peroxymonosulfate, accelerated hydrogen peroxide, peracetic acid, formaldehyde, sodium hypochlorite, or sodium hydroxide [18].

In this study, we carried out a disinfection efficacy study of four disinfectants on giant panda-derived FPV. We inoculated virus- and disinfectant-containing mixtures to FPV-sensitive cell lines to evaluate the efficacy with which the four different disinfectants inactivate FPV. The study may offer help to reduce the potential for viral spread via contaminated surfaces and prevent further outbreaks of FPV among the giant panda population.

## 2. Materials and Methods

### 2.1. Virus, Cells and Antibodies

F81 cells were kindly donated by Professor Zhang, College of Animal and Veterinary Sciences, Southwest University for Nationalities, cultured in RPMI-1640 medium (Biosharp, 69010400, Hefei, Anhui, China) containing 2% fetal bovine serum. Giant panda-derived FPV strain was preserved by the College of Veterinary Medicine, Sichuan Agricultural University.

The commercial antibodies used in the study are from EarthOx (FITC AffiniPure goat anti-rabbit IgG, E031220-01, Burlingame, CA, USA) and Proteintech (HRP Goat anti-Rabbit IgG, 15015, Chicago, IL, USA). Rabbit anti-FPV capsid polyclonal antibodies were prepared in this study. Sample collection was performed in a manner to minimize risk to the giant pandas and the environment.

#### Growth Kinetics

F81 cells were inoculated with giant panda-deprived FPV (100 μL of 100TCID_50_/mL). Incubate the cells at 37 °C with 5% CO_2_. The supernatants were collected at different times (12, 24, 36, 48, 60, 72, 96, 120 h.p.i), and the virus titers were determined via TCID_50_ assay. Briefly, F81 cells were plated on 96-well plates at 90% confluence. Virus supernatants were 10-fold serially diluted, and 100 μL of each was added to each well with eight replicates. The number of wells with visible cytopathic effect (CPE) was counted, and virus titers were calculated via the Reed–Muench method.

### 2.2. Disinfectants Tolerance Test for F81 Cells

The PAA (Zhongguang Decontaminant, 20210202, Chengdu, Sichuan, China), PVP-I (Huaxu Biotechnology Co., Ltd., 210318, Rizhao, Shandong, China), JM (Huaxu Biotechnology Co., Ltd., 21041201, Zhengzhou, Henan, China) and Virkon S (Kemu Pet Products Shanghai Co., Ltd., 2021010691, Shanghai, China) were prepared to working concentrations with RPMI-1640 medium containing 2% serum according to instructions. The disinfectant-containing medium solutions were filtered through a 0.22 μm filter (Biosharp, BS-QT-011, Hefei, Anhui, China) and were 10-fold serially diluted to 10^−1^–10^−3^. F81 cells monolayers were inoculated with each concentration with three replicates and inoculated with RPMI-1640 medium as negative control. Incubate the cells at 37 °C with 5% CO_2_.

### 2.3. Virucidal Assay

FPV was incubated at room temperature with working concentration of four disinfectants for 1 h, 2 h and 4 h. Then, then the mixtures were 10-fold serially diluted. F81 cells in 12-well plates were inoculated with the mixtures and inoculated with RPMI-1640 medium as negative control and FPV as positive control. Set three replicates for each group. Incubate the cells at 37 °C with 5% CO_2_.

### 2.4. Hemagglutination Assay (HA)

The hemagglutination assay (HA) was performed according to standard procedures. Briefly, two-fold dilutions of the disinfectant-containing medium were incubated with 1% porcine erythrocytes (diluted in PBS) in V-bottom, 96-well plates (NEST, 701211, Wuxi, Jiangsu, China). Plates were incubated for 2 h at 4 °C, after which hemagglutination titer was scored.

### 2.5. Immunofluorescence Assay (IFA)

F81 cells were seeded onto 14 mm glass cover slips in 24-well plates (NEST, 702001, Wuxi, Jiangsu, China). They were infected with FPV and disinfectant-treated supernatant. At 48 h post infection, cells were washed with PBST three times and treated with 4% paraformaldehyde (PFA) for 2 h at RT. The cells were then incubated in blocking buffer (PBS + 2% BSA + 0.5% Tween-20) containing rabbit anti-FPV (1:200) for 1 h at RT in dark. After three washing steps with PBST, cells were incubated with secondary antibody Goat anti-rabbit-FITC (1:500) in blocking buffer for 1 h at RT in dark. The cells were then washed five times with PBST. Cells were examined by fluorescence microscope (Olympus, Tokyo, Japan). 

### 2.6. Western Blot Analysis

F81 cells were infected with FPV- and disinfectant-containing medium. The infection was allowed to proceed for 48 h. Cell debris was pelleted for 3 min 12,000 rpm at 4 °C after three freeze–thaw cycles. Cleared lysates were harvested and kept at −80 °C. Samples of each lysate was separated by SDS-PAGE. The proteins were then blotted onto 0.2 μm polyvinylidene fluoride (PVDF) membranes by wet electrophoretic transfer. Membranes were washed three times in PBST, 5 min each, and incubated in blocking buffer (PBST + 3% BSA) for 2 h at RT. Membranes were successively incubated with primary antibody diluted in blocking buffer (FPV-positive serum, 1:100) overnight at 4 °C, followed by Horseradish Peroxidase (HRP)-conjugated Goat-anti rabbit IgG (1:4000). Between and after the incubations, the membranes were washed, thrice each time, with PBST. Finally, membranes were examined using SuperSignal West Pico PLUS (ThermoFisher Scientific Inc., Waltham, MA, USA) according to the manufacturer’s instructions.

### 2.7. Quantitative PCR

Viral DNA were extracted through extraction kit (R4173-02, Magen, Guangzhou, Guangdong, China). The relative qPCR was performed with the qPCR SYBR green master mix (Yeasen Biotechnology (Shanghai) Co., Ltd., 11201ES03, Shanghai, China). The primers are designed as follows: sense primer 5′-GGATCTGGGAACGGGTCTGGAG-3′ and antisense primer 5′-AAGTCTGCTTGAGTTTGCTGTGATTTC-3′.

### 2.8. Statistical Analysis

All statistical analyses were performed using the two-tailed Student’s *t*-test with GraphPad Prism 9 (GraphPad Software). *p* < 0.05 was considered statistically significant. 

## 3. Results

### 3.1. FPV Increased Exponentially in the First 48 h

**Figure 1:**
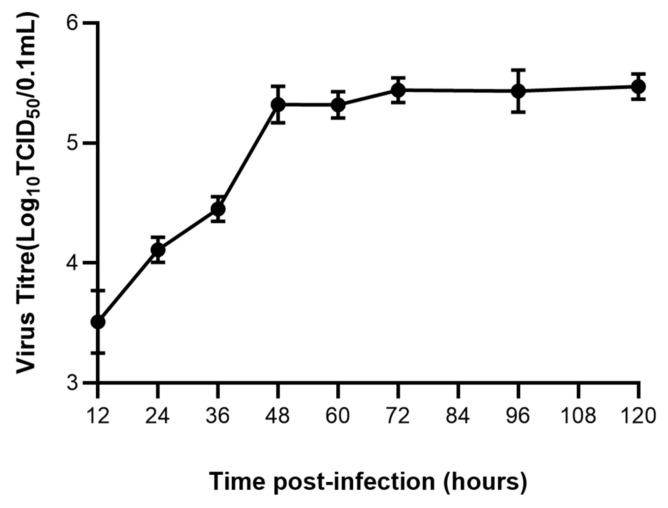
Growth kinetics of FPV in F81 cells.

The viral titer of the FPV strain propagated in F81 cells was determined at different time. The viral titer reached 10^−5.5^ TCID_50_/0.1 mL at 48 h (Figure 1).

### 3.2. Virkon S, JM and PAA Had Greater Effect on Cell Viability

**Figure 2:**
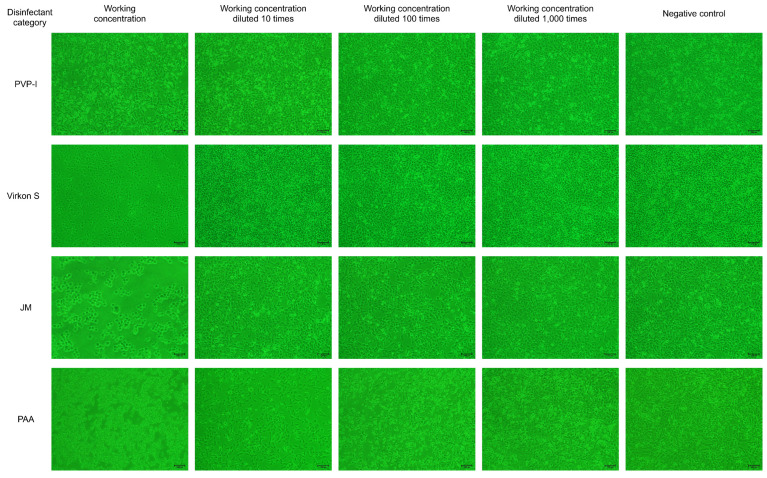
The disinfectant tolerance test for F81 cells (100×).

As results showed in Figure 2, PVP-I did not significantly affect cell viability at the working concentration, while the cellaggregation, and which eventually shed completely, appeared in F81 cells in the Virkon S- and JM-treated groups. The scenario also appeared in F81 cells in the PAA-treated group at the working concentration, even after a dilution of 100 times. The results indicated that Virkon S, JM and PAA will significantly affect cell viability at the working concentration, while PVP-I will not.

### 3.3. Verification of FPV Inactivation in F81 Cells 

**Figure 3:**
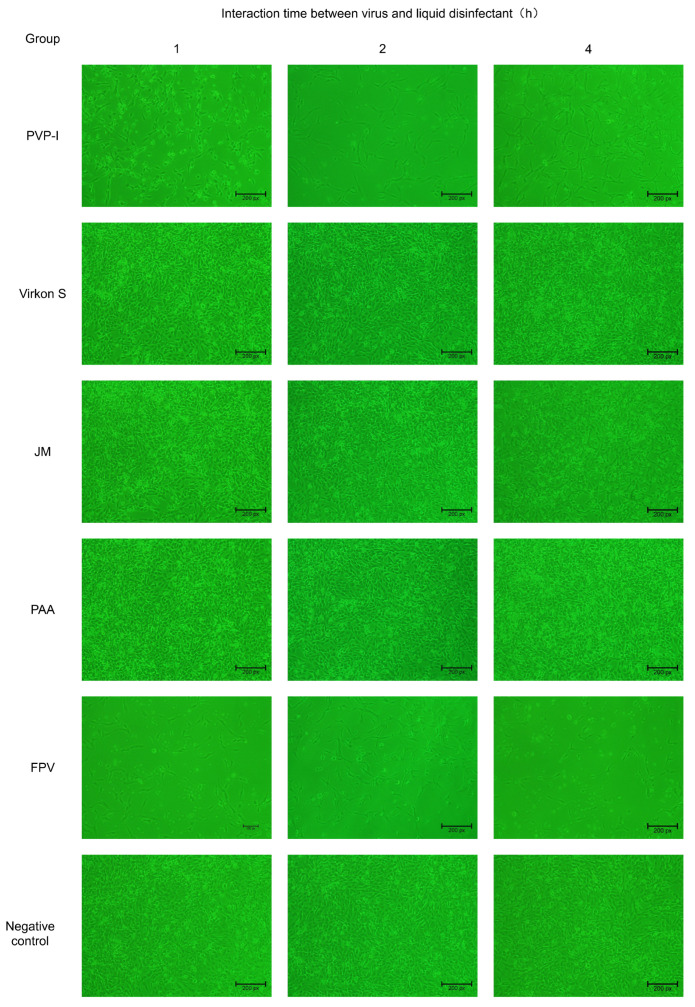
Verification of inactivation of FPV by four disinfectants in F81 cells (100×).

To confirm whether FPV has lost its infectivity after being inactivated by four disinfectants, the disinfected FPV was collected and inoculated in F81 cells. The results indicated no evident CPE was observed in the PAA-, Virkon S- and JM-treated groups, whereas CPE was observed in the PVP-I-treated group. The CPE that appeared in F81 cells of the PVP-I-treated group was the same as that in the positive control group, which is characterized by cell rounding, mitosis and shedding (Figure 3).

### 3.4. Efficacious Inactivation of FPV by PAA, Virkon S and JM

**Figure 4:**
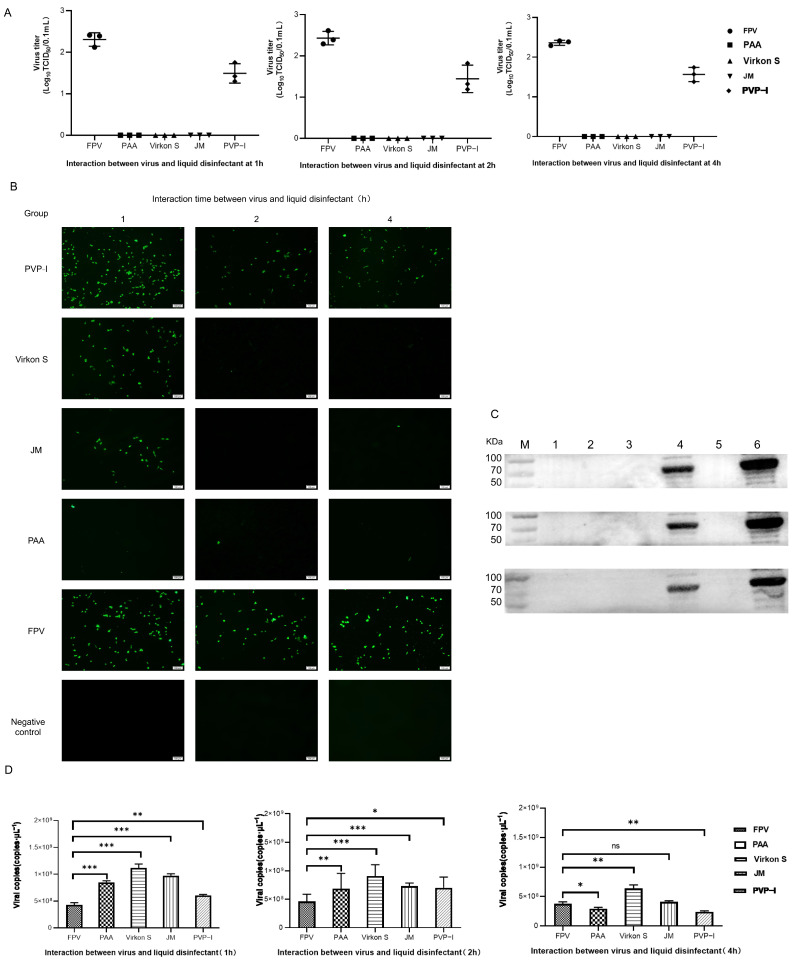
(**A**) Virus titration of FPV after different disinfectant treatments. (**B**) Virus proliferation changes after treatment with different disinfectants (100×). (**C**) Western blot analysis of VP2 protein of FPV. Lane 1, PAA; Lane 2, Virkon S; Lane 3, JM; Lane 4, PVP-I; Lane 5, NC; Lane 6, FPV. (**D**) Viral DNA copies after disinfectant treatment. Statistical analysis was performed by two-tailed Student’s *t*-test. Asterisks (*) indicate the statistical significance: * *p* < 0.05; ** *p* < 0.01; *** *p* < 0.001; ns, no significance.

**Figure 5:**
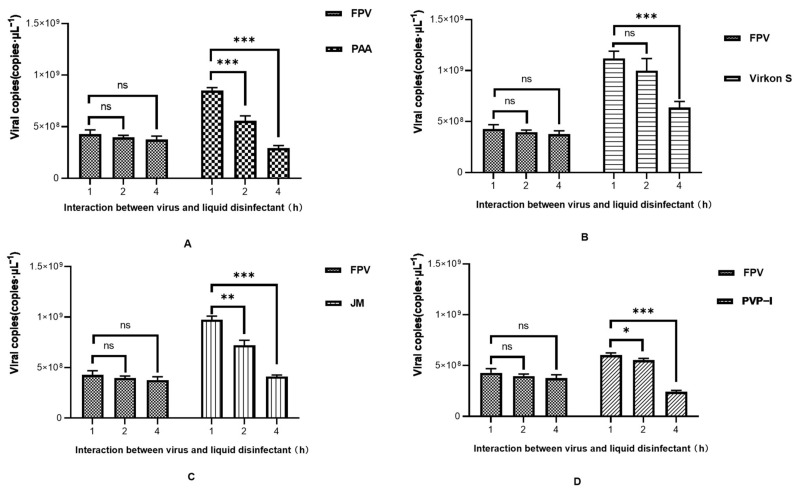
Time course study of viral DNA copies after disinfectant treatment. (**A**) PAA; (**B**) Virkon S; (**C**) JM; (**D**) PVP-I. Statistical analysis was performed by two-tailed Student’s *t*-test. Asterisks (*) indicate the statistical significance: * *p* < 0.05; ** *p* < 0.01; *** *p* < 0.001; ns, no significance.

As the results in Figure 4A show, FPV in the PAA-, Virkon S- and JM-treated groups showed no signs of propagation. Meanwhile, the viral titer of the PVP-I-treated group significantly decreased to 10^−1.5^TCID_50_/0.1 mL, compared with the titer of the positive control group (10^−2.2^TCID_50_/0.1 mL). We further conducted a detailed study to explore the approximate time of the effect of each disinfectant through IFA. As shown in Figure 4B, PAA, Virkon S and JM works within 1 h. The Western blot analysis also showed that PAA, Virkon S and JM can evidently inhibit the expression of VP2 (Figure 4C). To corroborate our observations, we measured the viral copies via qPCR in disinfectant-treated and FPV-infected F81 cells. In all treatments, the viral copies in the Virkon S group were higher than that of the control. In contrast, the viral copies in the PVP-I and PPA groups were lower than that of the control (Figure 4D). On the other side, the viral copies decreased over the time of incubation with the disinfectants (Figure 5). Taken together, PAA exhibited lower cytotoxicity and inactivation efficacy, which is significant.

### 3.5. PAA Had the Strongest Impact on HA Characterization

**Figure 6:**
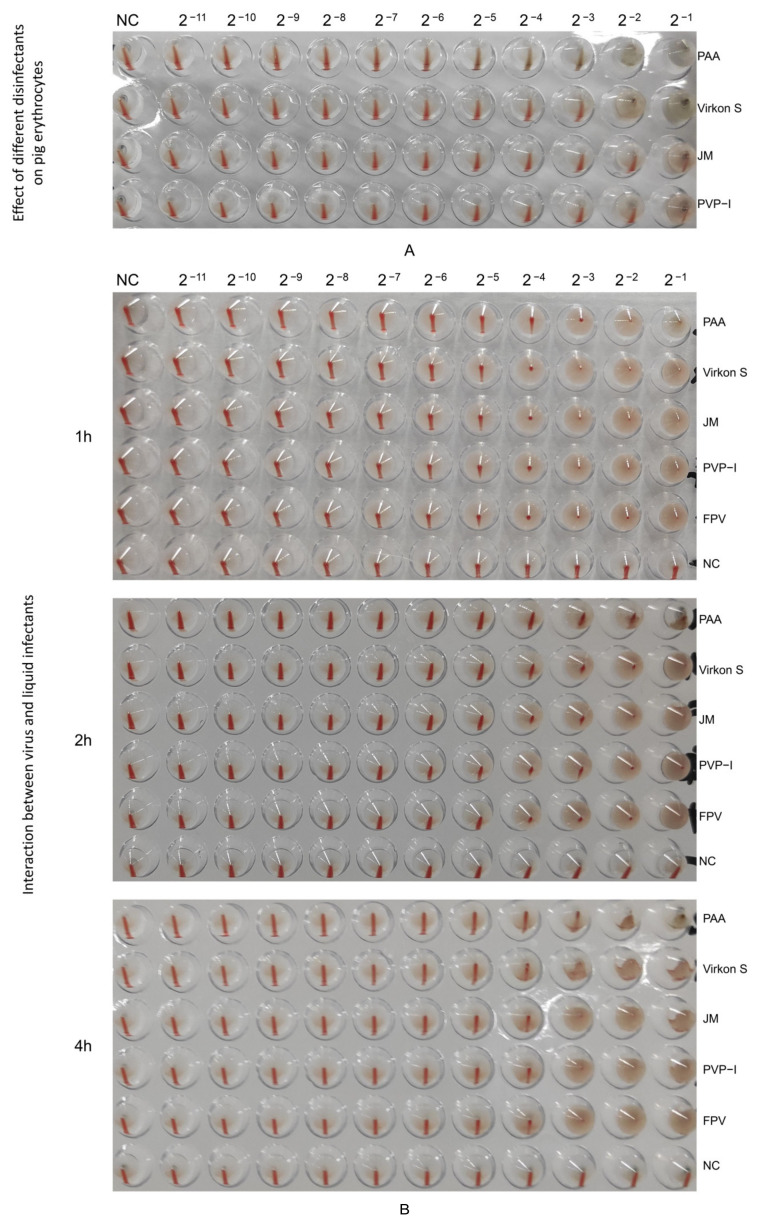
Determination of HA titers. (**A**) Effect of different disinfectants on pig erythrocytes. (**B**) Changes in hemagglutination potency of virus treated with different liquid disinfectants.

Firstly, we tested the effect of four disinfectants on 1% porcine erythrocytes, and the results showed that PAA and Virkon S did not exhibit inhibition at 2^−3^, and also JM and PVP-I at 2^−2^ (Figure 6A). An HA test was conducted with 1% porcine erythrocytes suspension to detect the HA titer of the wild FPV and the FPV after inactivation by disinfectants. The HA titer of PAA-treated FPV decreased by 1 titer within 1 h (Figure 6B). The HA titer of PAA-treated FPV decreased by 2 titers, while that of Virkon S-treated group decreased 1 titer within 2 h (Figure 6B), indicating PAA also had strong impact on the HA characterization of FPV.

## 4. Discussion

FPV, the most common carnivore protoparvovirus 1, is able to infect most domestic or wild carnivores [1]. In domestic cats, FPV can infect a broad spectrum of tissues and trigger a number of diseases, including fetal death after systemic infection in fetuses, ataxia and myocarditis after cerebellar or cardiac infections of neonatal cats [19]. The existence of FPV infection in giant pandas was firstly demonstrated with the clinical symptom of mild diarrhea in 2021 [15], and the phylogenetic analysis showed that the giant panda-sourced FPV isolate was grouped with other Chinese FPV strains. This report suggested that domestic or stray cats may have been the source of infection in giant pandas. Due to the increasing risk of FPV infection in giant pandas, better prevention and control are needed.

In this study, the efficacy of PAA, Virkon S, JM and PVP-I for the inactivation of FPV was evaluated in the laboratory. PAA is highly oxidizing and volatile, mainly affecting the loss of normal function of certain amino acids in the viral nucleocapsid, thus efficaciously inactivating the virus [20]. Virkon S, which mainly contains of potassium persulfate complex salt, has the advantages of being a stable, convenient and broad-spectrum disinfectant, and it can achieve the inactivation of the pseudorabies virus (PRV) within 30 min [21]. JM is common and mainly used for disinfection of a piggery environment and disinfection pools [22]. PVP-I is a broad-spectrum disinfectant that is efficacious against bacteria, fungi and viruses. The efficacy of PVP-I in killing microorganisms depends on the concentration of free iodine [23].

All four disinfectants can inactivate the virus to some extent, but none of them measured the inactivation of FPV. Due to the constraints of the host animal, giant pandas, this experiment was explored at the cellular level and was not practiced in the environment, which is somewhat flawed.

The expression level of the FPV VP2 protein was analyzed via Western blot. This protein accounted for 90% of the whole virus particles [24], and it can determine the coagulation characteristics and antigenicity of the virus and contained many antigen epitopes [25]. As a result, the analysis of the expression level of VP2 can directly reflect the amount of virus from the side. VP2 protein synthesis was greatly reduced after inactivation by PAA, Virkon S and JM within 1 h, and the inoculation of F81 cells failed to produce the corresponding CPE. According to the results of the HA, the HA titer of FPV after disinfectant treatment would decrease partially, and we assumed this may because the disinfectant only inhibits partial VP2 protein synthesis [26]. However, taking the results of IFA together, an efficacious disinfectant could still inhibit virus proliferation.

We observed the viral DNA copies at different times of disinfectants-treated FPV. In the beginning, the viral DNA copies in the four treatment groups were higher than that of the FPV group, which may be because the inhibition towards cells by disinfectants creates conditions conducive to the proliferation of FPV. Disinfectants not only affect viral protein synthesis, but also cell propagation. At first, disinfectants accelerate the progress of DNA replication, and then it might be easier to use organelles to synthesize nucleic acids after disinfectants have some effect on cells [27]. Different disinfectants exhibited different effects; for example, PVP-I had the weakest stimulating effect, but also the weakest inhibitory effected on virus proliferation. On the contrary, PAA had a slightly stronger stimulating effect than PVP-I, but it possesses the strongest inhibition, making it the most efficacious disinfectant.

In conclusion, PAA is a safe and efficacious disinfectant for FPV, providing a reference for the selection of disinfectants for the prevention and control of FPV.

## 5. Conclusions

The viral enteritis of giant pandas due to the giant panda-derived FPV has caused great harm to captive giant pandas. This study explored the efficacy of four disinfectants (PVP-I, PAA, JM, and Virkon S) on the giant panda-derived FPV for the first time. PAA is the most efficacious one, which could be used as a routine disinfectant in giant panda habitats. It can provide a reliable means of epidemic prevention for giant panda viral diseases, and it has great application potential in habitats.

## Data Availability

All data analyzed during this study are available from the corresponding author on reasonable request.
